# Arsenic Bioaccessibility of Realgar Influenced by the Other Traditional Chinese Medicines in Niuhuang Jiedu Tablet and the Roles of Gut Microbiota

**DOI:** 10.1155/2019/8496817

**Published:** 2019-12-17

**Authors:** Wenfeng Xu, Shuo Xu, Shanshan Zhang, Xuejun Wu, Pengfei Jin

**Affiliations:** Department of Pharmacy, Beijing Hospital, National Center of Gerontology, Assessment of Clinical Drugs Risk and Individual Application Key Laboratory, Beijing 100730, China

## Abstract

Niuhuang Jiedu tablet (NJT), a realgar (As_2_S_2_) containing Traditional Chinese Medicine (TCM), is a well-known formula. The safety of NJT is of growing concern since arsenic (As) is considered as one of the most toxic elements. NJT was demonstrated to be safer than realgar by our previous experiments and some other studies. The toxicity of realgar has been shown to be related to the amount of soluble or bioaccessible arsenic. In this study, the influences of the other TCMs in NJT on the bioaccessibility of arsenic from realgar, and the roles of gut microbiota during this process were investigated *in vitro*. Results showed that Dahuang (*Rhei Radix et Rhizoma*), Huangqin (*Scutellariae Radix*), Jiegeng (*Platycodonis Radix*), and Gancao (*Glycyrrhizae Radix et Rhizoma*) could significantly reduce the bioaccessibility of arsenic from realgar in artificial gastrointestinal fluids. Gut microbiota played an important role in decreasing the bioaccessibility of realgar because it was demonstrated to be able to absorb the soluble arsenic from realgar in the incubation medium. Dahuang, Huangqin, and Jiegeng could modulate the gut microbiota to enhance its arsenic absorption activity.

## 1. Introduction

Realgar, containing arsenic disulfide (As_2_S_2_) as the main component, has been used in Traditional Chinese Medicines (TCMs) for many centuries. The first medicinal use of realgar in China can be traced back to 200 B.C. in *Shen Nong Ben Cao Jing*, the first TCM book of China [1]. It has been applied for the treatment of external carbuncles, lumps, furuncles, boils, insect- and snake-bites, and internal parasitoses, convulsive epilepsy, and malaria [2]. In recent years, realgar has also been demonstrated to have beneficial therapeutic effects on acute promyelocytic leukemia and other human malignancies [3–8]. Niuhuang Jiedu tablet (NJT) is one of the most popular over-the-counter TCMs with hundreds of years of clinical application. According to the 2015 edition of Chinese Pharmacopeia (ChP), NJT is composed of realgar and seven other medicinal materials, including Rengong Niuhuang (*Bovis Calculus Artifactus*), Shigao (*Gypsum Fibrosum*), Dahuang (*Rhei Radix et Rhizoma*), Huangqin (*Scutellariae Radix*), Jiegeng (*Platycodonis Radix*), Bingpian (*Borneolum Syntheticum*), and Gancao (*Glycyrrhizae Radix et Rhizoma*). It is widely used as an antipyretic and detoxicate drug for sore swollen throat, periodontitis, gingivitis and mouth ulcer [2].

Arsenic exists in the trivalent and pentavalent forms and is a widely ranging and naturally occurring toxic element. A growing amount of evidence links arsenic exposure to increase cardiovascular disease, cancer, hematuria, and other forms of physical damage [9]. International Agency for Research on Cancer (IARC) Monographs has classified arsenic and inorganic arsenic compounds as carcinogenic hazards to humans [10]. Arsenic has been placed at the top of the Substance Priority List of the Agency for Toxic Substances and Disease Registry (ATSDR) since 1997 [11]. The quality and safety of realgar and realgar-containing TCMs are of growing concern. Realgar has been shown to cause acute or chronic toxic reactions in animals by some toxicological analyses [12–15]. As an adjuvant ingredient in NJT, realgar is considered to be important for good bioactivity of NJT because of its anti-inflammatory and analgesic effects [16]. It is unscientific and unreasonable to remove realgar from NJT just because it is a potential source of arsenic toxicity without considering its therapeutic effects and without the support of relevant experimental studies. To evaluate the risk of NJT, our previous experiments and some other studies demonstrated that NJT was safer than realgar, and components in NJT had synergistic detoxication effect on realgar [17–21].

Total content alone may lead to an overestimate of potential toxicity of realgar unless bioaccessibility is considered. Bioaccessibility represents the fraction of element that is released in digestive juices and may be considered as an indicator of maximal oral bioavailability. Realgar is poorly soluble in aqueous solutions due to its high intrinsic lattice energy, and only soluble arsenic in gastrointestinal fluids could be absorbed as the source of arsenic toxicity by the body [22]. Since realgar is commonly prescribed in Chinese medicinal formulae, the amount of dissolved arsenic leached from realgar-containing formulae could be affected by the coexisting herbal components, and therefore arsenic absorption and metabolism might be altered [1, 23]. However, the influences of the other TCMs in NJT on the solubility of toxic arsenic leached from realgar, and their synergistic interactions on dissolution rate of soluble arsenicals still lack system investigation.

The gut microbiota is very important for human health and is often considered as a “hidden organ” [24]. It is estimated that the microbes residing in or on human body slightly exceed the number of human cells [25], and gut bacteria encode at least 150 times as many genes as the human genome [26]. Under normal circumstances, human tissues and bacteria interact and maintain a functional balance [27]. Recently, the effect of gut microbiota on arsenic has been found to be an important factor affecting arsenic toxicity. Gut microbiota could decrease the arsenic load and protect the host from the liver toxicity of arsenic [28]. Either microbiome perturbation or absence could increase host arsenic bioaccumulation and toxicity [29]. However, the effect of human gut microbiota on the bioaccessibility of arsenic in realgar and NJT has rarely been reported and demands a detailed research.

Many atomic spectrometry techniques including graphite furnace atomic absorption spectrometry (GF-AAS), hydride generation atomic fluorescence spectrometry (HG-AFS), inductively coupled plasma atomic emission spectroscopy (ICP-AES), and inductively coupled plasma mass spectrometry (ICP-MS) have been used for trace elements analysis of all kinds of samples. ICP-MS, employed in this study, has been widely used for arsenic determination due to its low limits of detection, wide dynamic linear range, rapid detection, and few mass interference [30, 31]. This study aimed to systematically study the influences of the other TCMs in NJT on the bioaccessibility of arsenic from realgar in gastrointestinal fluids *in vitro*, and the roles of gut microbiota during the process, especially the effects of the other TCMs in NJT on the gut microbiota, and then on the bioaccessibility of arsenic from realgar.

## 2. Materials and Methods

### 2.1. Chemicals and Reagents

Realgar was purchased from Xi'an Yuelai Pharmaceutical Technology Co., Ltd. (Xi'an, China). *Bovis Calculus Artifactus*, *Gypsum Fibrosum*, *Rhei Radix et Rhizoma*, *Scutellariae Radix*, *Platycodonis Radix*, *Borneolum Syntheticum*, and *Glycyrrhizae Radix et Rhizoma* were supplied by Beijing Tongrentang Group Co., Ltd. (Beijing, China). *Rhei Radix et Rhizoma* and *Glycyrrhizae Radix et Rhizoma* were authenticated by Prof. Pengfei Jin as root and rhizoma of *Rheum palmatum* L. and root and rhizoma of *Glycyrrhiza uralensis* Fisch., respectively. All TCMs were tested according to ChP (2015) before the experiment, and the results showed that all the TCMs used were in accordance with the standards of ChP (2015).

Reference standard solution of arsenic (1000 mg/L) was purchased from National Institute of Metrology (Beijing, China). Electron pure BV-III grade nitric acid was provided by Beijing Institute of Chemical Reagents (Beijing, China). 10 *μ*g/L tuning solution with mixed elements (Ce, Co, Li, Tl, and Y) and internal standard mix (10 mg/L of Li, Sc, Ge, Y, In, Tb, and Bi) was obtained from Agilent Technologies (Santa Clara, USA). Pepsin, pancreatin, sodium hydroxide, potassium dihydrogen phosphate, hydrochloric acid, tryptone, peptone from soya, peptone, yeast extract, serum powder, liver extract powder (beef), beef extract, glucose, sodium chloride, soluble starch, L-cysteine hydrochloride, and sodium thioglycolate were provided by Sinopharm Chemical Reagent Co., Ltd. (Shanghai, China). Water was purified with a Millipore Milli-Q system (Millipore, Burlington, USA). All the other chemicals were analytical reagents. Glass vessels in this experiment were soaked in 10% (v/v) HNO_3_ and washed with ultrapure water before used.

### 2.2. Preparation of the Traditional Chinese Medicines

NJT was prepared according to ChP [2]. The weight ratio of realgar, *Bovis Calculus Artifactus*, *Borneolum syntheticum*, *Gypsum Fibrosum*, *Rhei Radix et Rhizoma*, *Scutellariae Radix*, *Platycodonis Radix*, and *Glycyrrhizae Radix et Rhizoma* was 1 : 0.1 : 0.5 : 4 : 4 : 3 : 2 : 1. Realgar was processed by “*Shui Fei*” method, which is a traditional grinding in water procedure. The resulting realgar powder was sifted by a 200-mesh sieve. *Rhei Radix et Rhizoma* was crushed and sieved using a sifter (100 meshes per inch). *Bovis Calculus Artifactus* and *Borneolum Syntheticum* were porphyrized and sifted with a 100-mesh sieve, respectively. *Gypsum Fibrosum*, *Scutellariae Radix*, *Platycodonis Radix*, and *Glycyrrhizae Radix et Rhizoma* were decocted with water (1 : 8) for 2 h, a process that was repeated twice. After filtration, the filtrate was combined and lyophilized. The resulting extract was grinded and sieved with a 100-mesh sifter. Mix all the above powders evenly. The prepared NJT was inspected and confirmed to meet the criterions of ChP. Each kind of TCM could be prepared separately according to the settings of experimental groups.

### 2.3. Artificial Gastrointestinal Fluid Extraction

The scheme used for grouping is shown in Table 1 with six samples in each group, respectively. The dosage of realgar in each group is equivalent to the amount of realgar in a single dose of NJT. The dosages of the other TCMs were designed according to the compatibility proportion of NJT. Artificial gastric and intestinal fluids were prepared as described in ChP [32]. The method for preparing artificial gastric fluid was as follows. Dilute 234 mL of hydrochloric acid to 1000 mL with water to obtain diluted hydrochloric acid solution. Mix 16.4 mL of diluted hydrochloric acid solution with 800 mL of water. Dissolve 10 g of pepsin in this solution and adjust the total volume to 1000 mL with water. The method for preparing artificial intestinal fluid was as follows. Dissolve 6.8 g of potassium dihydrogen phosphate in 500 mL of water and adjust pH to 6.8 with 0.1 mol/L of sodium hydroxide to obtain solution A. Dissolve 10 g of pancreatin with appropriate volume of water to get solution B. Mix solution A with solution B and adjust the total volume to 1000 mL with water.

Each sample was placed into a 200 mL conical flask containing 100 mL simulated gastric fluid. After being shaken at 150 rpm for 1 h at 37°C, the solution was filtered. The residue was rinsed three times using simulated gastric fluid and kept until the next extraction process. The filtrate was diluted to 200 mL with water to obtain the simulated gastric fluid extract. The remaining residue was transferred into a 200 mL conical flask to which 100 mL of simulated intestinal fluid was added. The solution was shaken at 100 rpm for 4 h at 37°C before filtration. The residue was rinsed three times with simulated intestinal fluid. Dilute the filtrate to 200 mL with water to gain the final simulated intestinal fluid extract. Blank samples were prepared and analyzed within the same batch of samples. All extracts were diluted ten folds with 10% (v/v) HNO_3_ before determination.

### 2.4. Fermentation Experiments

Fermentation experiments were operated as described in the literatures [28, 33] with some modifications. The composition of 1 L of general anaerobic medium (GAM, pH 7.2 adjusted by saturated sodium hydroxide solution) used in this study is 10 g of tryptone, 3 g of peptone from soya, 10 g of peptone, 5 g of yeast extract, 13.5 g of serum powder, 1.2 g of liver extract powder (beef), 2.2 g of beef extract, 3 g of glucose, 2.5 g of potassium dihydrogen phosphate, 3 g of sodium chloride, 5 g of soluble starch, 0.3 g of L-cysteine hydrochloride, 0.3 g of sodium thioglycolate. The GAM was sterilized at 121°C for 45 min in glass vessels before sample preparation.

Fresh fecal samples were collected from three Chinese male volunteers (25–33 years old). All volunteers were in good health and were not given any antibiotic treatment for at least the past three months. After collection, fecal samples were immediately stored in sterile anaerobic containers and processed within 1 h. Fecal slurries were prepared by diluting the mixed feces with GAM to obtain 5% (w/v) suspensions. The fecal suspensions were filtered with two layers of gauze. The collected filtered suspensions were incubated at 37°C in an anaerobic incubator for 24 h to serve as the fermentation inocula. Ten milliliters of fermentation inocula and the residue of simulated gastric fluid extraction were introduced into the sterilized 200 mL glass vessels, which were added with 90 mL of GAM and flushed with nitrogen gas for 20 min to create anaerobic condition. The mixtures were then fermented under anaerobic condition at 37°C for 24 h. Ten milliliters of the medium containing bacteria were taken and centrifuged at 4000 rpm for 10 min. One milliliter of the supernatant was diluted 10 times by 10% (v/v) HNO_3_ for further analysis. The bacterial pellets were weighed and incubated with 2 mL of HNO_3_ at 80°C for 4 h and then diluted by water to 20 mL for arsenic determination. The corresponding control of each experimental group was incubated according to the above method without gut microbiota in the medium.

### 2.5. Instrumentation and Operating Conditions

An Agilent 7500a ICP-MS (Agilent Technologies, Santa Clara, USA) was used for the determination of arsenic. Software ICP-MS ChemStation Rev. B. 03.02 was used for data acquisition. Operating parameters for ICP-MS analysis are given in Table 2. The equipment tuning was performed daily to assure responses of at least 7000 counts per second (cps) for Li, 12000 cps for Y and 9000 cps for Tl, and at most 2% for CeO/Ce and 3% for Ce^2+^/Ce.

### 2.6. Analytical Methods

The content assay method of realgar was based on ChP [2]. The analytical method for determination of arsenic in gastrointestinal fluid was established by our previous research [22]. The extracts (Section 2.2) and standard solutions were introduced into the ICP-MS nebulizer by a sample tube (1.02 mm, i.d.). The internal standard solution was obtained by diluting the internal standard mix (Section 2.1) with 10% (v/v) HNO_3_ to give a concentration of 0.1 mg/L as Li, Sc, Ge, Y, In, Tb, and Bi and injected online via an internal standard tube (0.19 mm, i.d.) throughout the data acquisition process. The amount of soluble arsenic (*μ*g/g) was calculated as follows: concentration of soluble arsenic in extracts (subtract extract blanks, *μ*g/mL) × dilution factors (mL)/amount of total arsenic.

### 2.7. Statistical Analysis

All experimental data are presented as mean ± SD. One-way analysis of variance (ANOVA) followed by Dunnett's test was used to evaluate differences among groups. Statistical analysis was performed using SPSS Statistics 17.0 software (SPSS Inc., Chicago, USA). For all analyses, a *p* value of <0.05 was defined as significant difference.

## 3. Results

### 3.1. Method Validation

The method was validated by characteristic indices including linearity, limit of quantitation (LOQ), precision, accuracy, and stability, which was detailed in our previous article [22]. The calibration curve, which was automatically generated by Agilent ChemStation software, was expressed by plotting the ratio of counts per second (cps) for As and the internal standard Ge (*Y*) versus the corresponding concentrations of arsenic (*X*) as *Y* = 0.0175*X* + 0.0031. The calibration curve showed good linearity in the range of 1–500 *μ*g/L for arsenic with a correlation coefficient (*r*) of 0.9999. The LOQ was also automatically generated by Agilent ChemStation software with a value of 0.0526 *μ*g/L. The precision of the method was evaluated by calculation of the relative standard deviation (RSD) obtained from six samples. RSD value (*n* = 6) of 4.13% indicated good precision of the method. Stability study showed that total arsenic in samples was stable at the condition of 4°C for 8 h, and the RSD was 3.78%. The accuracy was determined by the mean recovery of the test samples. The mean recovery yield for arsenic was 93.25% with RSD (*n* = 6) of 4.54%.

### 3.2. Results of Artificial Gastrointestinal Fluid Extraction

The content of As_2_S_2_ in realgar was 96.8%, which is in accordance with the value specified in ChP (≥90.0%). Therefore, the total amount of arsenic is 0.6785 g/g realgar weight. The amounts of soluble arsenic in simulated gastrointestinal fluid extracts of each group are listed in Table 3. There were no significant differences in the pH values of artificial intestinal juice or artificial gastric juice in each group (data not shown). The concentrations of soluble arsenic in simulated gastric and intestinal fluid extracts of group NJT were 35.1% and 30.0% lower than those of group realgar, respectively, and the total concentration of soluble arsenic was 33.4% lower than that of group realgar. The concentrations of soluble arsenic in simulated gastrointestinal juice extracts of group RBC and group RBS were not significantly different from those of group realgar. Compared with group realgar, the average content of total soluble arsenic in samples of group RGF increased significantly from 8017.7 *μ*g/g to 9059.0 *μ*g/g (13.0%). *Rhei Radix et Rhizoma* could strikingly reduce the amount of soluble arsenic leached from realgar by 39.3% in artificial gastric juice and 45.4% in artificial intestinal juice, respectively. *Scutellariae Radix* significantly inhibited the solubility of realgar with reductions of 43.2% in artificial gastric juice and 31.7% in artificial intestinal juice. When realgar was combined with *Platycodonis Radix*, the content of total soluble arsenic was 5648.7 ± 157.7 *μ*g/g, which accounted for 70.5% of the level of group realgar. When realgar was prepared in combination with *Glycyrrhizae Radix et Rhizoma*, reduction in arsenic extraction rate was caused in comparison with group realgar. Results of soluble arsenic levels of group NJT-BC, NJT-BS, and NJT-GF showed no statistical significant variation compared with group NJT.

### 3.3. Results of Fermentation Experiments

**Figure 1:**
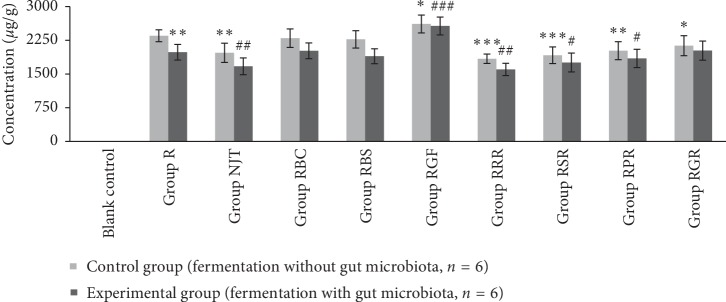
Soluble arsenic contents in the culture medium of each group (*n* = 6). Compared with the control group of group R: ^*∗*^*p* < 0.05, ^*∗∗*^*p* < 0.01, and ^*∗∗∗*^*p* < 0.001. Compared with the experimental group of group R: ^#^*p* < 0.05, ^##^*p* < 0.01, and ^###^*p* < 0.001.

**Figure 2:**
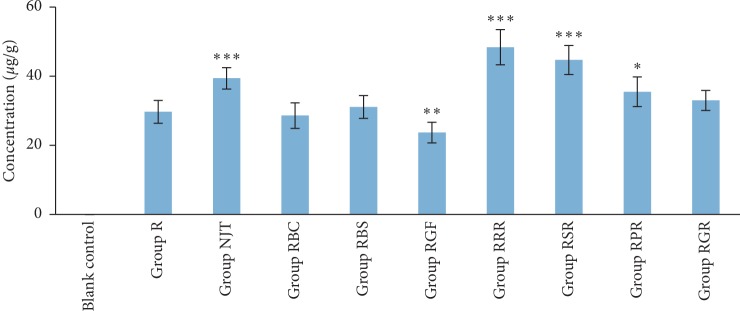
Total arsenic levels in bacterial cells of each group (*n* = 6). Compared with group R ^*∗*^*p* < 0.05, ^*∗∗*^*p* < 0.01, and ^*∗∗∗*^*p* < 0.001.

Figure 1 illustrates the comparison of soluble arsenic concentration in the medium of each group when fermentation with and without gut microbiota. After coincubation with gut microbiota, the arsenic content in the medium of each group decreased to different degrees.  Figure 2 shows the arsenic content in intestinal bacterial cells after fermentation in each group. As shown in Figure 1, *Bovis Calculus Artifactus* and *Borneolum Syntheticum* had no effects on the dissolution of arsenic from realgar, and *Rhei Radix et Rhizoma*, *Scutellariae Radix*, *Platycodonis Radix*, and *Glycyrrhizae Radix et Rhizoma* could reduce the dissolution quantity of arsenic in realgar under fermentation procedure without gut microbiota, which was consistent with the findings of artificial gastrointestinal fluid extraction experiment. When realgar was incubated with gut microbiota, the soluble arsenic concentration in the medium decreased significantly compared with the medium in which realgar fermented alone (Figure 1). Meanwhile, the arsenic levels in the bacterial cells of group realgar increased obviously, as shown in Figure 2. The concentration of arsenic in the cells of the gut microbiota was less than LOQ when fermented alone as the blank control. Compared with group realgar, there was no significant difference in the amount of arsenic accumulated in intestinal bacterial cells of group RBC and RBS. Arsenic content in the intestinal bacteria of group NJT was apparently higher than that of group R. The results of group RRR, RSR, and RPR were similar to that of group NJT.

## 4. Discussion

Because arsenic is a major toxic element and a potential carcinogen, the safety of sulfide form of arsenic as realgar (As_2_S_2_) and TCM compounds containing realgar have attracted extensive concerns. As a mineral medicine, realgar is an important ingredient and additive for the preparation of TCMs. It is believed that formulae consisting of multiple components would provide synergistic therapeutic effects and at the same time, subdue adverse effects mutually [34]. The bioavailability is a critical determinant of efficacy and toxicity of arsenical compounds. It was concluded that the toxicity of realgar could mainly be attributed to soluble arsenic since only the soluble portion of arsenic could be absorbed by bodies [16, 35].

This experiment systematically studied the influences of the other TCMs in NJT to the solubility of arsenic leached from realgar and their synergistic interactions on soluble arsenicals *in vitro*. The bioaccessibility of arsenic in realgar into the simulated gastrointestinal juice was 0.80% in this study, which was comparable with the values reported by other findings [18, 36, 37]. Arsenic concentrations in the two digestive juices varied extremely, which may be due to the differences in pH values of the two digestive juices. Elemental transfer to the bodies was essentially carried out by acidic gastric solution, and arsenicals are more soluble in acidic environment [38]. The compatibility of NJT could significant reduce the bioaccessibility of arsenic in realgar. This result was consistent with the findings of other comparative studies on bioavailability of realgar and NJT. *In vivo* studies indicated that coexisting herbs in NJT could significantly reduce the total blood arsenic in rats and alter the pharmacokinetic parameters of arsenicals from realgar [17, 19]. But the TCMs in NJT that could cause the above results were still obscure. The data of group RBC and group RBS were not different from that of group realgar, which indicated that *Bovis Calculus Artifactus* and *Borneolum Syntheticum* had no obvious effects on the amount of arsenic leached from realgar. There were no significant changes in the results of group NJT-BC and group NJT-BS compared with group NJT, which further demonstrated that *Bovis Calculus Artifactus* and *Borneolum Syntheticum* did not interfere with the reduced dissolution rate of realgar in this prescription. The combination of *Gypsum Fibrosum* and realgar could increase the content of total soluble arsenic by 13.0%. However, the data of group NJT-GF and group NJT showed no significant difference, indicating that *Gypsum Fibrosum* did not affect the soluble arsenic profile of NJT. *Rhei Radix et Rhizoma*, *Scutellariae Radix*, *Platycodonis Radix*, and *Glycyrrhizae Radix et Rhizoma* could be considered as the bases for reducing the bioaccessibility of realgar in NJT since these four herbs could decrease the amount of soluble arsenic leached from realgar by 39.3%, 43.2%, 30.9%, and 9.4% in artificial gastric solution, and by 41.3%, 39.4%, 29.5%, and 9.7% in artificial intestinal solution, respectively. The pH value of gastrointestinal juice in each group was not considered as one of the factors that affected the experimental results because there were no significant differences in the pH values of the artificial intestinal juice or artificial gastric juice in each group. As reported in the literature, some herbal ingredients of realgar-containing TCMs showed detoxication effects by reducing the solubility of toxic arsenic [35]. *Rhei Radix et Rhizoma*, *Scutellariae Radix*, *Platycodonis Radix*, and *Glycyrrhizae Radix et Rhizoma* were proved to have detoxication effects on realgar in rats by our previous metabonomics study [19]. One of the underlying mechanisms may be related to the reduced bioaccessibility of arsenic in realgar. The chemical components in the four herbs that can reduce the bioaccessibility of arsenic in realgar should be further explored. Standardized and reliable *in vitro* models can be used to evaluate the bioaccessibility of toxic elements from TCMs, and bioaccessibility is only one factor determining bioavailability and toxicity. Further relevant *in vivo* experiments on animals are extremely required.

The gut microbiota plays an important role in xenobiotic biotransformation and can affect the toxic effects of xenobiotics. The interaction between the gut microbiota and arsenic has become an important factor to understand the toxicity of arsenic [28]. Realgar exposure has been demonstrated to disrupt the metabolism of intestinal microbial communities by our previous studies [21]. The microbiomes of humans and mice were shown to have the ability to metabolize arsenic by previous *in vitro* studies [39–42]. In this *in vitro* study, when co-fermented with realgar, gut microbiota was shown to be able to absorb or accumulate arsenic and thus reduce the soluble arsenic in the medium. This finding was consistent with another previous study, in which gut microbiota has also been demonstrated to absorb arsenic when co-incubation with sodium arsenite as the source of arsenic [28]. Gut microbiota could also bioaccumulate arsenic *in vivo*, for both antibiotic-treated and germ-free mice were seen to excrete less arsenic in stool and accumulate more arsenic in organs [29].

The content of arsenic in the medium of co-incubation of group RRR and gut microbiota was less than that of group R and gut microbiota. This result could not precisely indicate that *Rhei Radix et Rhizoma* could increase the arsenic absorption activity of gut microbiota, for *Rhei Radix et Rhizoma* itself could reduce the content of soluble arsenic from realgar in the medium. Therefore, the contents of arsenic in intestinal bacterial cells of each group were further determined. The arsenic contents in intestinal bacterial cells of group RRR, RSR, RPR, and NJT were significantly higher than that of group R, which indicated that *Rhei Radix et Rhizoma*, *Scutellariae Radix* and *Platycodonis Radix* in NJT could increase the absorption activity of gut microbiota to arsenic *in vitro*. Gut microflora-associated alterations to achieve homeostasis of the gut ecosystem and pathophysiology of the host can be assessed to better understand the complex mechanisms of TCMs [43]. According to literatures, *Rhei Radix et Rhizoma* had modulation effects on gut microbiota. The extract of *Rhei Radix et Rhizoma* could prevent hepatic inflammation induced by acute alcohol intake by modulating the gut microbiota [44]. TCM formula Rhubarb Peony Decoction containing *Rhei Radix et Rhizoma* was confirmed to regulate gut microbiota to restore the gut homeostasis to treat ulcerative colitis [45]. The supplementation of *Rhei Radix et Rhizoma* could enhance host mucosal innate immune homeostasis by modulating intestinal epithelial microbiota in goat kids [46]. However, the regulatory effects of *Scutellariae Radix* and *Platycodonis Radix* on gut microbiota were rarely reported. Further experiments are needed to study the chemical components in *Rhei Radix et Rhizoma*, *Scutellariae Radix*, and *Platycodonis Radix* that can regulate the gut microbiota and to investigate the mechanisms by which the three herbs increase the absorption activity of gut microbiota to arsenic.

## 5. Conclusion

The safety of NJT has attracted great attention since arsenic is a major toxic element and a potential human carcinogen. In the present work, *Rhei Radix et Rhizoma*, *Scutellariae Radix*, *Platycodonis Radix*, and *Glycyrrhizae Radix et Rhizoma* in NJT were confirmed to be able to reduce the bioaccessibility of arsenic from realgar *in vitro*. Gut microbiota could accumulate arsenic and therefore decrease the bioaccessibility of arsenic in realgar. *Rhei Radix et Rhizoma*, *Scutellariae Radix*, and *Platycodonis Radix* could increase the absorption activity of gut microbiota to arsenic *in vitro*. Further appropriate *in vivo* studies are required to determine the bioavailability and potential toxicity of realgar in NJT.

## Figures and Tables

**Table 1:** Grouping scheme of this experiment.

	Group name												
	Blank	R	RBC	RBS	RGF	RRR	RSR	RPR	RGR	NJT	NJT-BC	NJT-BS	NJT-GF
Realgar (R, 0.1 g)		✓	✓	✓	✓	✓	✓	✓	✓	✓	✓	✓	✓
*Bovis Calculus Artifactus* (BC, 0.01 g)			✓							✓		✓	✓
*Borneolum Syntheticum* (BS, 0.05 g)				✓						✓	✓		✓
*Gypsum Fibrosum* (GF, 0.4 g)					✓					✓	✓	✓	
*Rhei Radix et Rhizoma* (RR, 0.4 g)						✓				✓	✓	✓	✓
*Scutellariae Radix* (SR, 0.3 g)							✓			✓	✓	✓	✓
*Platycodonis Radix* (PR, 0.2 g)								✓		✓	✓	✓	✓
*Glycyrrhizae Radix et Rhizoma* (GR, 0.1 g)									✓	✓	✓	✓	✓

Note: ✓, corresponding TCM included.

**Table 2:** ICP-MS operating parameters.

Parameter	Value
RF power	1350 W
RF matching	1.7 V
Sampling depth	7.0 mm
Spray chamber	Quartz cooled to 2.0°C
Peristaltic pump speed	6 rpm
Carrier gas	0.96 L/min
Makeup gas	—
Interface cones	Ni
Acquisition mode	Spectrum analysis
Number of points per mass	3
Integration time per point	0.1 s
Number of repetition	3
Monitored elements for calculations	^75^As and ^72^Ge
Uptake speed	12 rpm
Uptake time	45 s
Stabilization speed	6 rpm
Stabilization time	65 s

**Table 3:** Concentrations of soluble arsenic in simulated gastrointestinal fluid extracts of each group (mean ± SD, *n* = 6).

Group name	Gastric fluid extract		Intestinal fluid extract		Total extract	
	Content (*μ*g/g)	Extraction rate (%)	Content (*μ*g/g)	Extraction rate (%)	Content (*μ*g/g)	Extraction rate (%)
Group R	5411.7 ± 244.7^#^	0.54	2606.0 ± 124.9^#^	0.26	8017.7 ± 267.3^#^	0.80
Group NJT	3513.5 ± 129.9^*∗∗*^ (↓35.1%)	0.35	1824.7 ± 72.4^*∗∗*^ (↓30.0%)	0.18	5338.2 ± 123.8^*∗∗*^ (↓33.4%)	0.53
Group RBC	5339.2 ± 273.1^#^	0.53	2682.0 ± 131.0^#^	0.27	8021.2 ± 376.3^#^	0.80
Group RBS	5540.9 ± 282.7^#^	0.55	2578.4 ± 131.8^#^	0.26	8119.3 ± 384.5^#^	0.81
Group RGF	6123.3 ± 314.5^*∗∗#*^ (↑13.1%)	0.61	2935.7 ± 132.8^*∗∗#*^ (↑12.7%)	0.29	9059.0 ± 321.7^*∗∗#*^ (↑13.0%)	0.91
Group RRR	3283.7 ± 160.3^*∗∗*^ (↓39.3%)	0.33	1423.0 ± 58.6^*∗∗#*^ (↓45.4%)	0.14	4706.6 ± 191.3^*∗∗#*^ (↓41.3%)	0.47
Group RSR	3076.0 ± 138.0^*∗∗#*^ (↓43.2%)	0.31	1779.1 ± 69.1^*∗∗*^ (↓31.7%)	0.18	4855.1 ± 183.0^*∗∗#*^ (↓39.4%)	0.49
Group RPR	3737.5 ± 110.8^*∗∗*^ (↓30.9%)	0.37	1911.2 ± 89.1^*∗∗*^ (↓26.7%)	0.19	5648.7 ± 157.7^*∗∗*^ (↓29.5%)	0.57
Group RGR	4904.7 ± 220.9^*∗#*^ (↓9.4%)	0.49	2338.0 ± 105.4^*∗∗#*^ (↓10.3%)	0.23	7242.7 ± 256.9^*∗∗#*^ (↓9.7%)	0.72
Group NJT-BC	3599.3 ± 179.0^*∗∗*^ (↓33.5%)	0.36	1941.0 ± 81.1^*∗∗*^ (↓25.5%)	0.19	5540.3 ± 227.0^*∗∗*^ (↓30.9%)	0.55
Group NJT-BS	3728.9 ± 168.0^*∗∗*^ (↓31.1%)	0.37	1821.2 ± 62.8^*∗∗*^ (↓30.1%)	0.18	5550.1 ± 137.2^*∗∗*^ (↓30.8%)	0.56
Group NJT-GF	3324.9 ± 142.7^*∗∗*^ (↓38.6%)	0.33	1756.7 ± 84.4^*∗∗*^ (↓32.6%)	0.18	5081.6 ± 190.3^*∗∗*^ (↓36.6%)	0.51

Compared with group R: ^*∗*^*p* < 0.01, ^*∗∗*^*p* < 0.01, and ^#^*p* < 0.05 versus group NJT.

## Data Availability

The data used to support the findings of this study are available from the corresponding author upon request.
